# Tuning magnetic anisotropy in Co–BaZrO_3_ vertically aligned nanocomposites for memory device integration†

**DOI:** 10.1039/c9na00438f

**Published:** 2019-09-30

**Authors:** Bruce Zhang, Jijie Huang, Jie Jian, Bethany X. Rutherford, Leigang Li, Shikhar Misra, Xing Sun, Haiyan Wang

**Affiliations:** School of Electrical and Computer Engineering, Purdue University West Lafayette Indiana 47907-2045 USA hwang00@purdue.edu; School of Materials Engineering, Purdue University West Lafayette Indiana 47907-2045 USA

## Abstract

Ferromagnetic nanostructures with strong anisotropic properties are highly desired for their potential integration into spintronic devices. Several anisotropic candidates, such as CoFeB and Fe–Pt, have been previously proposed, but many of them have limitations such as patterning issues or thickness restrictions. In this work, Co–BaZrO_3_ (Co–BZO) vertically aligned nanocomposite (VAN) films with tunable magnetic anisotropy and coercive field strength have been demonstrated to address this need. Such tunable magnetic properties are achieved through tuning the thickness of the Co–BZO VAN structures and the aspect ratio of the Co nanostructures, which can be easily integrated into spintronic devices. As a demonstration, we have integrated the Co–BZO VAN nanostructure into tunnel junction devices, which demonstrated resistive switching alluding to Co–BZO's immense potential for future spintronic devices.

## Abstract 2

Ferromagnetic nanostructures with tunable, strong anisotropic properties are highly desired for their potential integration into spintronic devices.

## Graphical abstract

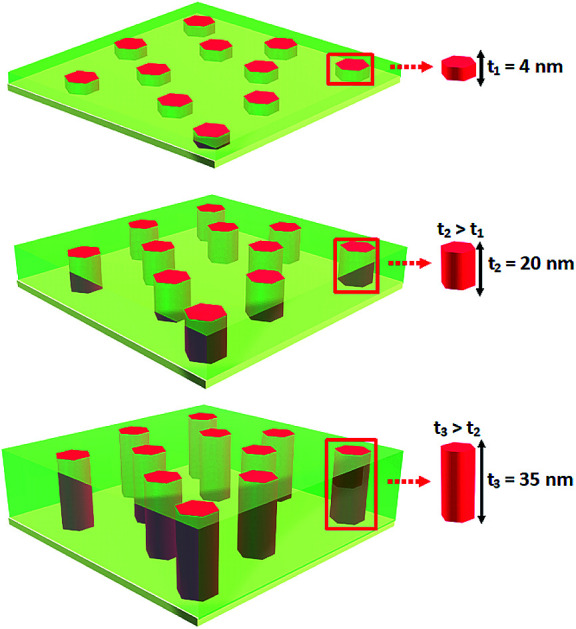

## Introduction

Spintronics^1^ cover a wide range of devices that operate *via* electron spins, such as neuromorphic memory,^2^ MRAM,^3–5^ Spin Torque Transfer (STT) devices,^5,6^*etc.* In spintronics, ferromagnetic materials are of great interest due to them being primary materials in spintronic devices. Taking a typical group of spintronic devices as an example, magnetic tunnel junctions have received great interest due to their potential for application in high performance memory devices and various other applications.^7–10^ Magnetic tunnel junctions typically consist of ferromagnetic films that play pivotal roles in resistive switching.^11^ To obtain higher resistive switching ratios and create more responsive magnetic tunnel junctions, extensive work has been done on tuning and fabricating ferromagnetic materials. To this end, two approaches to improve tunnel junction performances for low energy and high frequency applications include adjusting the coercive field of the ferromagnetic materials^12–16^ and integrating anisotropic ferromagnetic films.^17^ In the second approach, various ferromagnetic materials, such as SmCo_5_,^18^ CoFeB,^19–21^ Fe–Pt,^22^ and Pd/Co layered structures,^23^ have been explored by using strain^24,25^ and patterning^26^ to achieve their anisotropic magnetic properties. For example, CoFeB and Fe–Pt^19–22^ have shown anisotropic physical properties with certain thicknesses, while others^18,23^ require numerous layers or patterning to achieve anisotropic magnetization. Overall, techniques and modifications to tune the perpendicular anisotropy materials have shown success, but they require either stringent deposition conditions or post-processing techniques.

Vertically aligned nanocomposites (VANs) are self-assembled nanocomposite thin films with nanopillars (phase 1) embedded in a matrix (phase 2) allowing for tunable anisotropic physical properties without extensive processing and patterning requirements and thickness constraints,^27,28^ which allows the single-step growth of a wide range of 2-phase materials. In addition, VANs are of particular interest due to their versatile material combinations toward enhanced or new physical properties. For example, oxide–oxide VAN systems are widely reported to achieve highly desirable physical properties such as low field magnetoresistance,^29,30^ exchange bias,^31–33^ and multiferroic properties.^34^ Their unique physical properties, especially their tunable magnetic anisotropy, are of particular importance to magnetic tunnel junctions. Very recently, the development of metal–oxide based VAN systems^35–38^ extends ferromagnetic material candidates for VAN designs from oxides to metals allowing for achieving stronger ferromagnetic properties. Despite the challenges in the co-growth of metal–oxide due to the largely different growth conditions and possible oxidation of the metal, several metal–oxide VAN structures have been successfully achieved by pulsed laser deposition (PLD) with careful growth condition control. Some examples are Ni–CeO_2_,^35^ Ni–Ba_0.8_Zr_0.2_Y_3_,^36^ Co–BaZrO_3_ (BZO),^37^ Fe–La_0.5_Sr_0.5_FeO_3_,^38^ Au–BaTiO_3_,^39^ and Au–TiO_2_.^40^ All of the films are metal nanopillars in an oxide matrix and several systems have shown very strong anisotropic physical properties with tunable coercive fields, ideal for integration into spintronic devices.

**Fig. 1:**
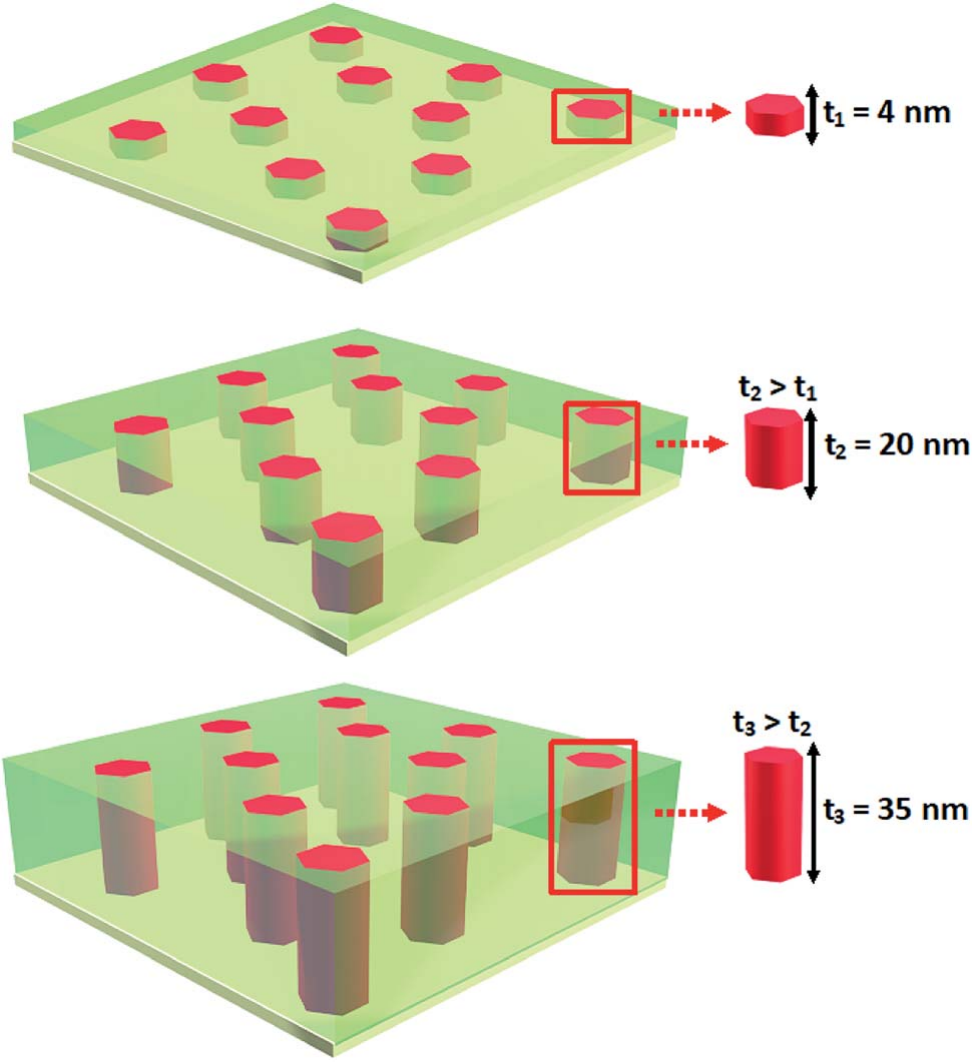
Schematic showing the different thicknesses investigated to determine how the thickness and, in extension, the aspect ratio between the thickness of the film and the diameter of the Co pillars affect the physical properties.

In this work, Co–BZO VAN thin films with well controlled nanopillar shape anisotropy (*i.e.*, the aspect ratios of the Co nanopillars) has been demonstrated with the goal of achieving tunable magnetic anisotropy and tunable coercive field strength of magnetic nanostructures. Previous work has shown tuning the physical properties *via* altering the deposition frequency,^37^ while this work demonstrates tuning the physical properties, *e.g.*, magnetic anisotropy, *via* tuning the aspect-ratio of the Co nanopillars. This study also allows the investigation of the growth mechanism of metal–oxide VANs. Such a tunability was achieved by controlling the film thickness from 4 nm to 35 nm, as shown in Fig. 1, with the thinnest being smaller than the diameter of the Co nanopillars and the thickest being significantly larger than the diameter of the Co nanopillars. The ferromagnetic properties in both in-plane and out-of-plane were subsequently measured and compared with the shape anisotropy in the Co-nanopillars. Finally, different from previous work, the Co–BZO thin films in this work were grown significantly below 100 nm in thickness, for the purpose of Co–BZO VAN integration into a spintronic device. This device demonstrates resistive switching properties under an applied magnetic field which highlights the potential of the Co–BZO VAN towards integrated tunnel junction devices.

## Experimental

The Co–BZO (Co–BaZrO_3_) target was prepared following the standard solid-state target processing steps. Initially, the BaZrO_3_ target was prepared *via* conventional powder pressing and sintering methods using BaCO_3_ and ZrO_2_ powders. Next, the BaZrO_3_ target was crushed and further ground into powder, and then combined with Co powders at a molar ratio of BZO : Co of 60 : 40 and sintered at 1100 °C in a flow of Ar/H_2_. The Co–BZO nanocomposite thin films were grown on SrTiO_3_ (STO) (001) *via* a PLD system (KrF laser, 248 nm) with varying thicknesses of 4 nm, 20 nm, and 35 nm. The depositions were performed at a frequency of 5 Hz under high vacuum conditions (<1 × 10^−6^ Torr) at 750 °C with a target-to-substrate distance of 5 cm.

The crystal structure and nanostructure of the films were studied by X-ray diffraction (XRD) (Panalytical X'Pert X-ray diffractometer), transmission electron microscopy (TEM), scanning transmission electron microscopy (STEM), and energy-dispersive X-ray spectroscopy (EDX) (FEI Talos-200X). The magnetization properties of all the samples were measured both in-plane and perpendicular to the thin film surface using a magnetic property measuring system (MPMS, Quantum Design). The electrical properties of the multilayer structure were measured using Au contacts deposited *via* PLD on the film using a physical property measuring system (PPMS, Quantum Design).

## Results and discussion

**Fig. 2:**
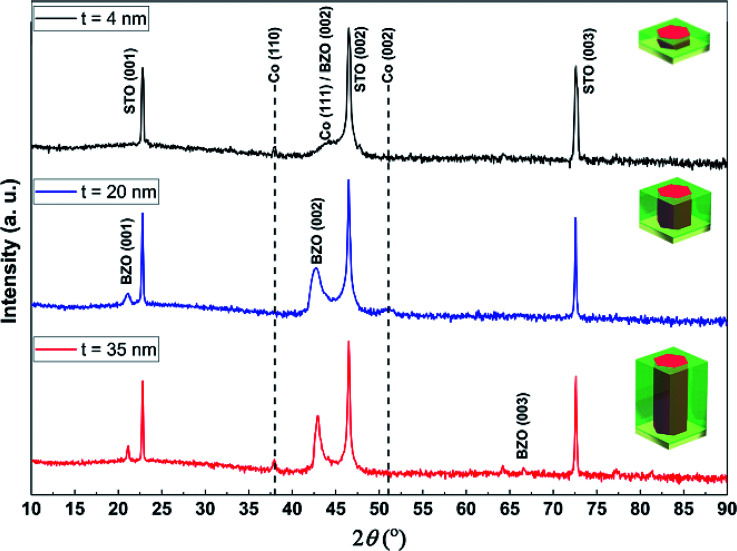
Typical *θ*–2*θ* XRD scan of the different films deposited with different thicknesses with the corresponding schematic to illustrate the aspect ratio.

Microstructure characterization by XRD was first conducted to analyze the growth orientation and growth morphology of the Co–BZO nanocomposite thin films with different thicknesses (4 nm, 20 nm and 35 nm). *θ*–2*θ* XRD scans are shown in Fig. 2 for all Co–BZO films. BZO (00*l*) peaks can be identified in the 20 nm and 35 nm films, but not in the 4 nm sample. The lack of BZO (00*l*) peaks in the 4 nm sample is possibly due to the ultrathin film and a small amount of BZO in the film. BZO (002) is located at around 42.81° with a corresponding *d*-spacing of 2.11 Å. All the Co peaks match well with the face centered cubic Co PDF card (PDF #15-0806), with the Co (111) peak (reference 2*θ* = 44.216, *d* = 2.0467 Å) in all samples, with the Co (200) peak (reference 2*θ* = 51.522, *d* = 1.7723 Å) in the 20 nm sample, and with the Co (110) peak (reference 2*θ* = 37.927, *d* = 1.2532 Å) in the 35 nm sample. The Co (111) peak is located at 43.96° with a corresponding *d* spacing of 2.06 Å for the 4 nm film only, indicating a tensile out-of-plane strain (0.68%) for Co due to the larger *d*-spacing of BZO (2.11 Å). For the 35 nm sample, the BZO (002) peak intensity is the highest, indicating better crystallinity of BZO in the 35 nm sample, and the Co (110) peak has the highest intensity indicating a preferred Co growth direction of Co (110) as the film thickness increases.

**Fig. 3:**
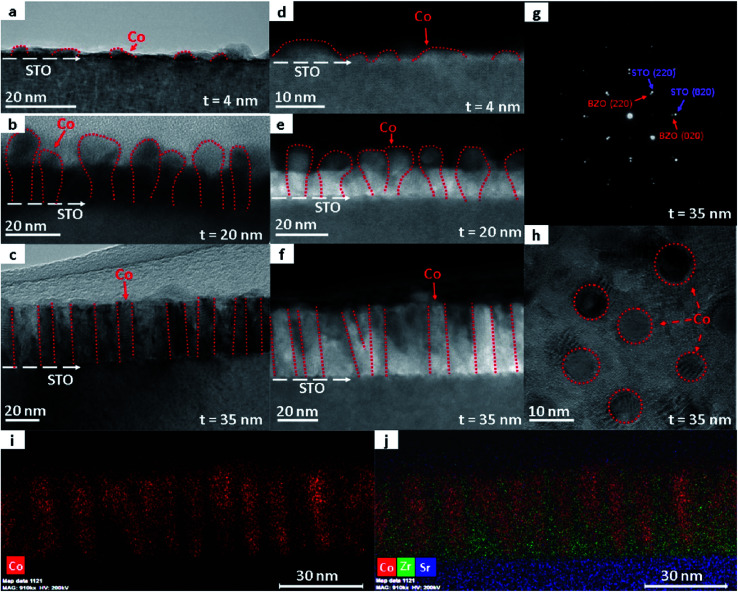
Nanostructure study of the Co–BZO samples grown with different thicknesses. (a–c) TEM images of the 4 nm, 20 nm, and 35 nm thick samples verifying the thicknesses. (d–f) STEM images of the 4 nm, 20 nm, and 35 nm thick samples. (g) Selected area electron diffraction (SAED) patterns of the 35 nm thick sample with a (h) selected plan view area sample for the 35 nm thick sample. EDS images were taken with a (i) Co spectrum map and an (j) element map.

To further verify the composition and investigate the epitaxial quality of the Co–BZO samples, a TEM study was conducted for all the samples with varying thickness. In Fig. 3a–c, low magnification cross-sectional TEM images of the 4 nm, 20 nm, and 35 nm samples are shown. The 4 nm sample presents island growth with the islands being Co and with BZO as the matrix, which is consistent with the XRD data and the EDX data shown in Fig. S1a.† As the film continues to grow, as seen in the 20 nm film, the film exhibits Co nanopillars with the top portion protruding through the surface of the film. In contrast, the 35 nm film exhibits a more uniform growth throughout the thickness of the film with more ordered Co regions in the Co–BZO nanocomposite. The structural tuning from Co islands to Co nanorods to uniform nanopillar growth arises from the overall interactions between the metal and the oxide adatoms on the substrate. Initially, the Co adatoms nucleate as islands, as seen in the 4 nm sample, due to the fact that the Co and BZO adatoms tend to coalesce due to their different surface energies (2.595–3.19 J m^−2^, 1.185 J m^−2^, and 1.26 J m^−2^ for Co, BZO, and STO, respectively)^41–44^ with Co having a higher surface energy when compared to STO. As the growth continues, since BZO has a lower surface energy than STO, it grows in a layer-by-layer fashion. Thus, as the Co island continues to grow, the BZO layer growth continues forming a 2-phase nanocomposite film between the two, as seen in the 20 nm sample. As the film continues to grow thicker, the BZO layer becomes more prominent and forms a smooth 2-phase nanocomposite with Co nanopillars well embedded in the oxide matrix, as seen in the 35 nm sample. Fig. 3d–f show the corresponding STEM images of the films obtained in the high angle annular dark field (HAADF) mode to further highlight the Co nanostructures in the BZO film. To further verify the growth quality, a typical diffraction pattern of the Co–BZO thin film, shown in Fig. 3g, was taken that further shows that cubic BZO grew epitaxially on the STO substrate. To acquire a rough distribution of the Co pillars, a plan view of the 35 nm sample was taken and is shown in Fig. 3h. The plan-view image shows a regular distribution of Co pillars in the BZO matrix with the pillars having an average diameter of 8 nm. For verifying the phase separation of Co and BZO, EDX images of the 35 nm sample are shown in Fig. 3i and j, which show that Co nanopillars are well separated from the BZO matrix. Overall, the thickness variation results in the structure anisotropy as proposed in the Co–BZO system, with the thinnest having just Co islands with small BZO layers and the thickest, the 35 nm sample, having a film with a more uniform thickness with regular Co pillars.

**Fig. 4:**
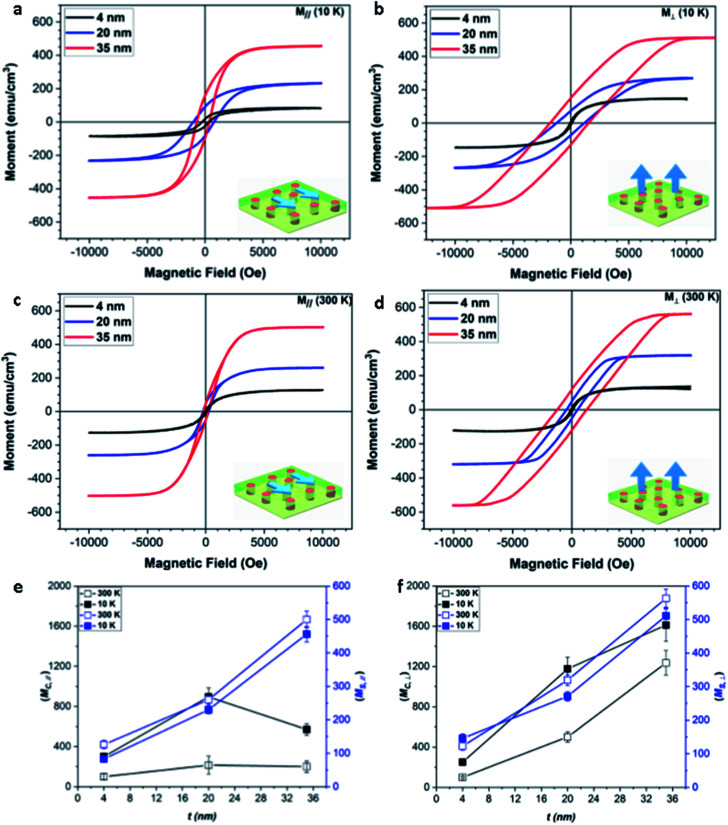
Magnetic study of the different films at (a and b) low temperatures with in-plane and out-of-plane applied fields, and at (c and d) room temperatures with in-plane and out-of-plane applied fields. Further plotting was done on the different coercive fields and magnetic saturations done for (e) in-plane and (f) out-of-plane applied magnetic fields at low and room temperatures.

To investigate how the shape anisotropy affects the magnetic properties of the Co nanostructures in Co–BZO, the magnetic responses of all the samples were measured using a VSM in MPMS. Initially, the *M*–*H* response was measured at 10 K and 300 K in both the in-plane and out-of-plane directions, as shown in Fig. 4a and c and Fig. 4b and d, respectively. It is important to note that the unit on the *y* axis is emu cm^−3^ and the volume used is the entire film as opposed to the volume of only the Co regions. Thus, the actual magnetization values of the Co regions could be much higher than the ones plotted. The overall shape and saturation values of the magnetization data agree well with previous work on Co–BZO grown on STO.^35^ In the in-plane direction (*i.e.*, parallel to the film surface), it shows a saturation magnetization of 83, 230, 456 emu cm^−3^ at 10 K and 126, 260, 500 emu cm^−3^ at 300 K for the 4 nm, 20 nm, and 35 nm films, respectively. Additionally, compared to the in-plane properties, the out-of-plane direction (*i.e.*, perpendicular to the surface) shows much stronger magnetic properties, with saturation magnetization of 146, 271, and 511 emu cm^−3^ at 10 K and 123, 319, 563 emu cm^−3^ at 300 K for the 4 nm, 20 nm, and 35 nm films, respectively, showing comparable saturation values from low to room temperature. The very similar *M*_S_ values are possibly attributed to the weak temperature dependence of *M*_S_ in Co from 10 K to 300 K and the strong strain coupling along the vertical oxide–metal interfaces in the samples. Furthermore, in the out-of-plane direction, an obvious increase in coercivity occurs with increasing thicknesses at 10 K with values of 250 Oe, 1175 Oe, and 1612 Oe for the 4 nm, 20 nm, and 35 nm samples, respectively, and at 300 K with values of 100 Oe, 500 Oe, and 1235 Oe for the 4 nm, 20 nm, and 35 nm samples, respectively.

As the thickness of the Co–BZO layer increases, the ferromagnetic properties of the samples also vary. The coercive field and saturation magnetization increase with increasing thickness. For coercive field specifically, the trend is due to the change in the grain size and domain size. Specifically, if each Co nanostructure is a single magnetic domain when the film reaches magnetic saturation, larger Co nanopillars or nanoparticles will require larger magnetic fields to saturate. As the Co pillars grow in height, the domain sizes increase. This subsequent increase in the domain size causes the applied magnetic field required to align the magnetization to increase. In addition, as the temperature increases from low temperature to room temperature, the coercive field decreases dramatically, especially for the thinner samples, which is typical for ferromagnetic materials.

**Fig. 5:**
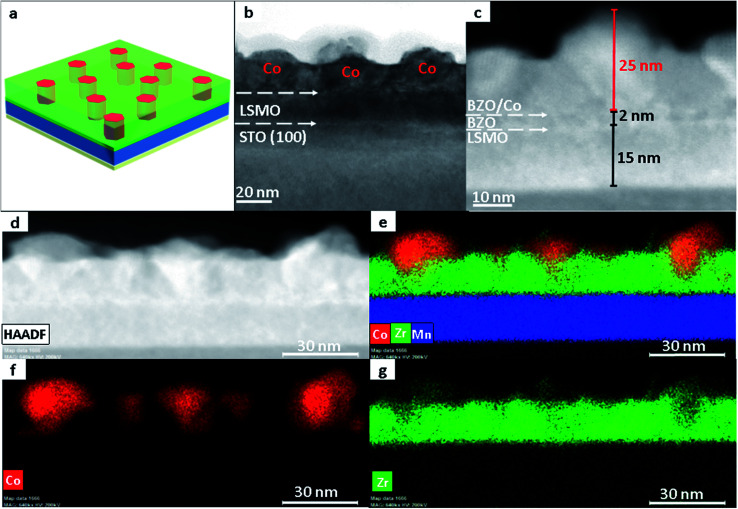
Nanostructure study of the device with a (a) schematic of the device and its corresponding (b) TEM image and (c) STEM image. In addition, the (d) HAADF images were taken with the (e–g) corresponding elemental mappings.

**Fig. 6:**
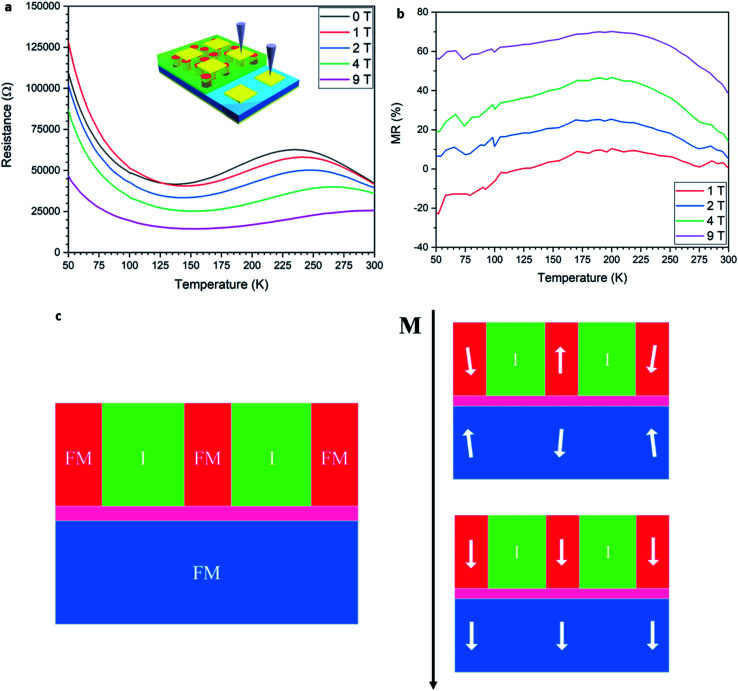
Magnetoelectric properties of the device with (a) *M vs. R* with varying applied fields with its corresponding (b) MR ratios. (c) A schematic of how the spins align in the ferromagnetic layers as magnetic fields increase.

The Co–BZO nanocomposite film was further integrated into a device for achieving resistive switching and magnetoresistance properties. A Co–BZO sample with the thickness of around 20 nm was selected because of the combination of its soft magnetic properties and its magnetic anisotropy. Fig. 5a shows the proposed device structure, and TEM, STEM, and EDX of the tunnel junction are shown to verify the growth quality. The 2 nm BZO layer serves as an insulating layer between the two ferromagnetic layers of Co–BZO and La_0.7_Sr_0.3_MnO_3_ (LSMO). BZO was selected owing to its lattice matching of the oxide matrix of Co–BZO. The Co–BZO layer shows a similar nanostructure to that seen when grown directly on STO. LSMO was selected as the bottom electrode layer due to both its semi-metal conducting behaviour, allowing for a bottom electrode, and its room temperature ferromagnetic behaviour. This configuration allows for the ferromagnetic coupling between the top Co–BZO layer and the lower LSMO, semi-metal layer, thus allowing for electrical tunnelling across the interface to be controlled by an applied magnetic field. Since BZO is an effective insulator, temperature will also play a crucial role in the electron transport properties of the multilayer stack. As seen in Fig. 6a, the device shows resistance switching with an applied magnetic field that is perpendicular to the film. The resistance was measured by taking *R*–*T* measurements from both the top electrode on Co–BZO and the bottom electrode on the LSMO film. Magnetoresistance (MR%) was calculated using eqn (1).^45^ The MR values show correlation between the magnetic field and the resistance with a peak of 10, 25, 47, and 70% under 1 T, 2 T, 4 T, and 9 T, respectively, at a temperature of 200 K.1
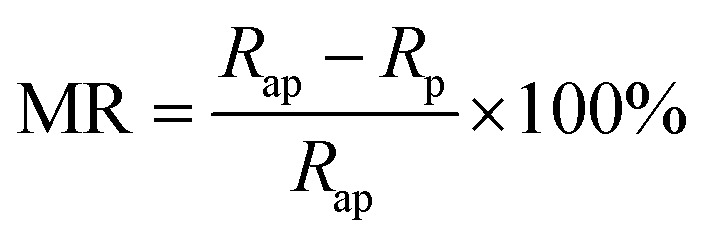


As the magnetic field increases, the MR value increases due to the interaction as shown in Fig. 6c, where at zero applied field, the magnetic orders for both the top Co–BZO and the bottom LSMO layers are not aligned. Increasing the magnetic field to 1 T results in Co–BZO reaching magnetic saturation and the LSMO to not be strongly aligned in the out-of-plane direction resulting in higher resistances than at zero applied field as seen in Fig. 6a and b. As the applied magnetic field continues to increase, the magnetic domains become more ordered resulting in the ferromagnetic layers to be aligned in the same direction. The increase in ordering allows for electrons to pass through the barrier more readily, which decreases the resistance and causes a change in magnetoresistance. This is seen by the subsequent increases in the MR% as the magnetic field increases from 1 to 9 T in Fig. 6b. The direct correlation between MR and applied magnetic field, wherein as the field increases the MR increases, demonstrates the successful fabrication of a ferromagnetic device that allows for the tuning of resistance *via* magnetic field. Thus, the growth of Co–BZO VANs on top of a ferromagnetic semi-metal and insulator resulted in a MR change with changing magnetic field. This shows their successful integration into a spintronic device and shows the potential of metal–oxide VANs for device applications and, especially as the tunability of the Co–BZO is further investigated, allowing for eventual integration into tunnel junction devices.

## Conclusion

In summary, a strong anisotropic ferromagnetic material, Co–BZO nanocomposite thin films, has been demonstrated with tunable coercive field, and these thin films have been integrated into a spintronic device. Their strong magnetic anisotropy and coercive field tuning properties address the need for low energy, high density, and energetically stable devices. The successful integration of such a Co–BZO nanostructure into a device structure opens new avenues for metal–oxide VANs and their future integration into devices towards low power tunnel junction devices and many other spintronic devices.

## Conflicts of interest

There are no conflicts to declare.

## Supplementary Material

NA-001-C9NA00438F-s001
