# Supplementary material for: Tuning magnetic anisotropy in Co–BaZrO3 vertically aligned nanocomposites for memory device integration
Source: Nanoscale Adv. 2019 Sep 30;1(11):4450–8. doi: 10.1039/c9na00438f (PMC9417828; doi:10.1039/c9na00438f)
Supplement: NA-001-C9NA00438F-s001 [file NA-001-C9NA00438F-s001.pdf]

Electronic Supplementary Material (ESI) for Nanoscale Advances.
This journal is © The Royal Society of Chemistry 2019

s1

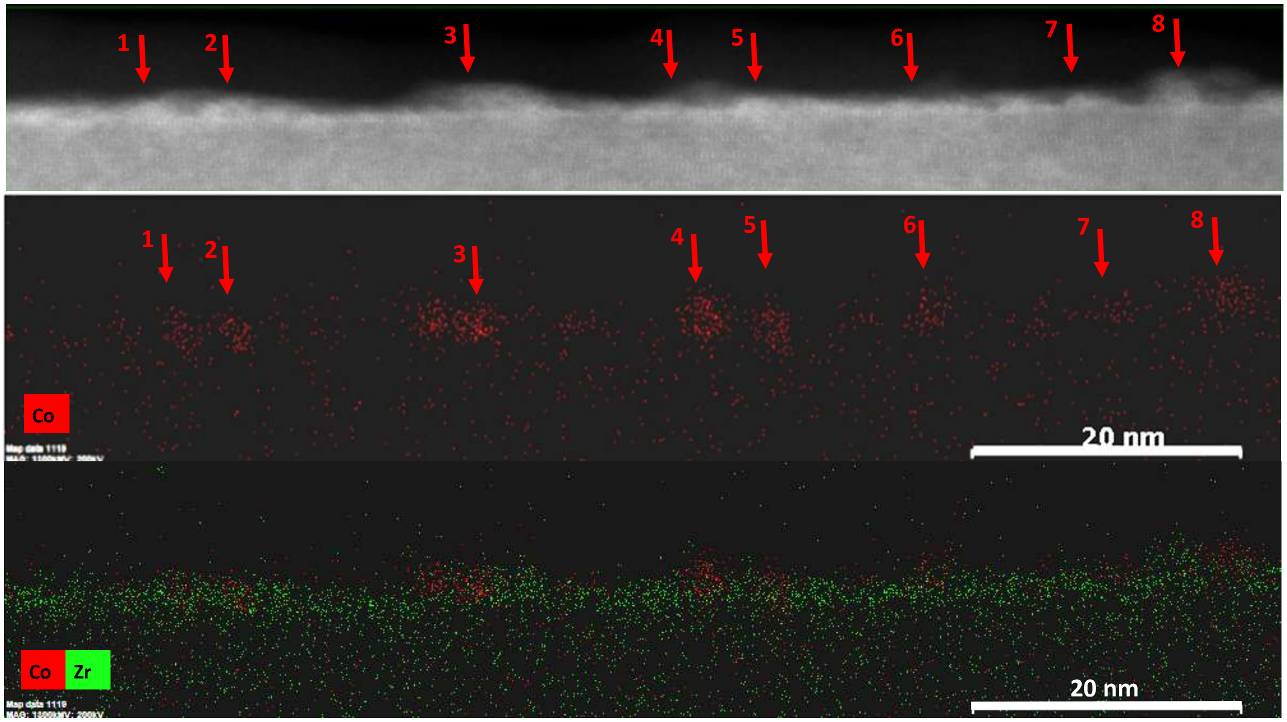

Figure S1. STEM with an EDS mapping verifying the Co islands with BZO region on the 4 nm thick sample with the numbers in the STEM referring to their respective regions in the EDS mapping
